# Calorie labelling and other drivers of takeaway food choices

**DOI:** 10.1136/bmjnph-2025-001268

**Published:** 2025-08-12

**Authors:** Laura Cornelsen, Oana Adelina Tanasache, Cherry Law, Tahrima Choudhury, Richard D Smith, Steven Cummins

**Affiliations:** 1 Department of Public Health, Environments and Society, London School of Hygiene & Tropical Medicine, London, UK; 2 School of Social Sciences and Global Studies, Faculty of Arts and Social Sciences, The Open University, Milton Keynes, UK; 3 Department of Agri-Food Economics & Marketing, University of Reading, Reading, UK; 4 Medical School, University of Exeter, Exeter, UK

**Keywords:** Dietary patterns

## Abstract

**Background:**

Frequent consumption of out-of-home (OOH) foods, including takeaways, is linked to higher energy intake and poorer diet quality. In April 2022, calorie labelling was mandated in England for large OOH businesses to support healthier choices. This paper aimed to explore knowledge and use of calorie labelling when ordering takeaway food online and other factors influencing food choices, overall and by sociodemographic characteristics.

**Methods:**

A cross-sectional survey of 1040 takeaway consumers in England from an OOH purchase panel assessed knowledge and awareness of calorie labelling legislation, self-reported impact on online takeaway choices, and key drivers of takeaway consumption. Data were analysed using descriptive statistics, logistic regressions and thematic analysis.

**Results:**

Over 27% of respondents ordered takeaways once or more per week. Respondents aged 35 years and older were less likely (OR 0.28–0.52, p<0.05), while those with obesity were more likely (OR 2.01, p<0.001) to report frequent takeaway purchases. Women were more knowledgeable about the recommended energy meal content than men (OR 2.06, p<0.001), yet only 15% of respondents knew the guideline amount. Awareness of calorie labelling regulations was 63% overall, but was less likely in middle socioeconomic groups (OR 0.56–0.63, p<0.05). During past online orders, 23% noticed calorie labels; of these, 26% reduced food calories ordered, and 10% reduced drink calories ordered. Taste and price were key drivers of takeaway choices, while health and sustainability were less influential. Strong support emerged for healthier menu options and traffic light labelling to help improve the healthiness of takeaway orders.

**Conclusion:**

Given the limited reported impact, calorie labels may only benefit a minority. As meal healthiness was ranked as a relatively less important driver for takeaway choices, further policies are required to complement calorie labelling in promoting healthier food environments.

**WHAT IS ALREADY KNOWN ON THIS TOPIC**

- Out-of-home (OOH) foods tend to be lower in nutritional quality. Although frequent consumption is linked to poorer dietary health, there remains limited understanding of the factors driving OOH purchases and how interventions like calorie labelling promote healthier choices.

**WHAT THIS STUDY ADDS**

- This study highlights that online takeaway ordering is more prevalent among younger adults and individuals living with obesity. While awareness of England’s calorie labelling regulations was high, actual use of calorie information when selecting food or drink was low. Taste and price were indicated as the most important drivers of choice, outweighing health and sustainability considerations.

**HOW THIS STUDY MIGHT AFFECT RESEARCH, PRACTICE OR POLICY**

- Calorie labelling alone has a limited impact but may still play a valuable role as part of broader strategies to address obesity and promote healthier eating environments.

## Introduction

 Improving diet remains a key public health priority in England where obesity rates among adults and children are persistently high.^1^ While obesity has complex causes, frequent out-of-home (OOH) food consumption may be one contributing factor.^2 3^ OOH foods, including those from restaurants, fast food chains, coffee shops, vending machines and canteens,^4^ are often higher in energy and fat compared with home-cooked meals.^4^ This may explain the link between more frequent OOH eating, poorer diet quality and higher body weight.^5 6^

Consumption and drivers of OOH food consumption remain poorly researched despite their significant role in dietary choices. In England, 70% of households purchased OOH foods in 2021/22, representing a quarter (25%) (author calculations using Living Cost and Food Survey data 2021–22 release; Total Restaurant and Hotels category excluding accommodation related expenditure and expenditure on alcoholic drinks) of total food and non-alcoholic drink expenditure.^7^ Data from 2008 to 2012 UK National Diet and Nutrition Survey (NDNS) showed that adults who ate out (27%) or got takeaways (20%) at least weekly consumed 75–104 kcal and 63–87 kcal more, respectively, than adults who had these foods less frequently.^8^

In April 2022, calorie labelling for large OOH food businesses (>250 employees), including online deliveries and takeaways, was mandated in England. The policy has increased calorie labelling at point-of-choice from 21% to 80%,^9^ but awareness of labelling is low. In a study of 330 OOH outlets in England, only 32% of customers noticed calorie labels after the policy was implemented, of which only 19% reported using them. The study also found no overall effect (or by sociodemographic subgroups) from calorie labels on calories ordered or consumed.^10^

Evidence from review-level studies on the effectiveness of calorie labelling in OOH settings remains mixed. A recent systematic review and meta-analysis—drawing primarily on studies from the USA (n=15), but also including the UK (six), Ireland (one), France (one), and Canada (one)—found that calorie labelling led to an average reduction of 11 kcal in calories selected and 35 kcal consumed.^11^ An earlier review, also largely US-based, reported an 18 kcal reduction in calories selected.^12^ However, two other reviews found no overall impact on calories ordered or consumed.^13 14^ Most of these reviews highlighted effect heterogeneity by venue type, study design and whether the studies were conducted in real-world versus laboratory settings.

Berry *et al*, based on two restaurant field experiments, suggested that calorie labelling may be effective for health-orientated consumers but could lead others to order higher-calorie foods, perceiving them as greater value for money or tastier.^15^ Another study found adults who reported using calorie labels in restaurants (27%) had better overall diets compared with those who reported not noticing labels.^16^ These studies suggest that calorie labels might primarily benefit consumers who are already health motivated.

Calorie labels are only effective if consumers understand what calories are and know how to use this information. Poor health literacy and numeracy have been identified as barriers to interpretation.^17^ In England, it is recommended that main meals contain around 600 kcal,^18^ but to our knowledge, awareness of this has not been studied. While labelling legislation in England mandates the statement ‘Adults need around 2000 kcal a day’ alongside calorie labels on menus, energy needs vary by age, sex, and physical activity levels. Thus, calorie label use requires a high level of understanding of what calories are, how these are spent, and how they fit with daily energy needs.

This paper aimed to explore the knowledge and use of calorie labelling when ordering takeaway food online and its self-reported impact overall and by sociodemographic characteristics, utilising data from a survey of 1040 adult takeaway consumers in England. To understand takeaway choices further, we also examined consumption frequency and asked respondents to rank drivers of takeaway choices and possible policies to encourage healthier takeaways.

## Methods

Data were collected via an online survey following a choice experiment on calorie labelling of online takeaway food.^19^ The survey covered six themes: (1) takeaway frequency; (2) knowledge of recommended calorie content of a meal; (3) awareness of calorie labelling; (4) noticing calorie labels and their perceived impact; (5) ranking of drivers of takeaway and home-cooked meal choices (healthiness, price, taste, preparation/delivery time, portion size and low carbon footprint); and (6) ranking of five policies encouraging healthier takeaway choices (higher prices for less healthy items, smaller portions, physical-activity calorie equivalent labels, traffic light labels, more healthier alternatives). This was followed by an optional free text question asking respondents to suggest any other policies that they feel would encourage healthier takeaway choices. The exact wording of the questions, answer options and outcome coding are shown in online supplemental table S1. Ethical approval for the study was provided by the London School of Hygiene & Tropical Medicine Ethics Committee (ref 27959).

### Participant sampling and recruitment

The online survey was distributed in November 2022 to a sample of approximately 1600 individuals who are part of the Kantar Worldpanel out-of-home purchase panel. Kantar’s Worldpanel is a commercial data, insights and consulting company (www.kantar.com/uki/about). They operate a consumer panel in Great Britain consisting of approximately 30 000 households who record take-home purchases of food and drinks. The panel is stratified by household size, number of children, occupational socioeconomic status, geographical region, and age group. A representative sub-panel of approximately 6000 respondents also records OOH purchases from which the sample for this study was drawn. The sample was restricted to adult respondents who lived in England and had ordered takeaway food online (via a website or an app) at least once in the previous 12 months. Respondents with vegetarian, vegan or otherwise restricted diets were excluded.

### Sociodemographic characteristics

Sociodemographic characteristics of the respondents were provided by Kantar and are collected on an annual basis. These include region of residence (London, South, East, North, Midlands), gender (male or female), age (<35, 35–44, 45–54, 55–64, ≥65) and occupational socioeconomic status (SES) according to the National Readership Survey (AB – higher & intermediate managerial, administrative, professional occupations; C1 – supervisory, clerical and junior managerial, administrative and professional, C2 – skilled manual workers, D – semi-skilled and unskilled manual workers, E – state pensioners, casual and lowest grade workers, unemployed with state benefits only).^20^ In addition, we asked for respondents’ height and weight to calculate body mass index (BMI) using the standard formula (kg/m²) and categorised based on healthy weight/underweight (BMI <25), overweight (BMI 25–29) and obese (BMI >30).

### Analytical approach

Outcome variables were built from the survey questions (full list in online supplemental table S1 or table 2, column 3) and coded predominantly as binary. One outcome (knowledge of recommended calorie content for a meal) was coded as ordinal with three categories. Distribution of responses in the full sample was analysed descriptively. Multivariate logistic regression models were then used to explore associations between outcomes and sociodemographic characteristics. All sociodemographic variables described above entered the model as categorical variables.

As height or weight was missing for 189 respondents (18%), we assessed whether missingness was random with respect to the outcome. This was tested using logistic regression of a binary variable describing BMI missingness (1 – missing, 0 – otherwise) against each outcome and remaining covariates (results available from the authors). For completeness and comparisons, BMI was included in all models, although where this was not deemed as missing at random (five outcomes), we warrant caution in the interpretation. Quantitative analyses were conducted in Stata SE/18.0.^21^

The free text question ‘Are there any other policies which you feel would encourage healthier takeaway food choices?’ had 455 responses overall. Of these, 65% (298/455) were deemed as valid responses. The remaining 35% (157/455) were responses such as ‘None’, ‘Don’t know’ and ‘No’. We used thematic analysis with an inductive approach to analyse free text responses. After familiarising ourselves with the text (TC, LC), responses were coded (TC) and themes generated (TC), which were then reviewed and finalised (TC, LC).

## Results

In total, 67% (n=1040) of the total sample invited completed the survey, with a 2% dropout rate. Demographic characteristics are shown in table 1. The sample had more female than male respondents (68%), predominantly aged between 35 and 55 years (>50%) and largely in SES group C1 (45%). Just over half of the sample were living with overweight (28%) or obesity (35%). Compared with Kantar’s estimates of online takeaway consumers in England (Kantar’s Worldpanel OOH purchase panel, online food delivery, 52 w/e 17 April 2022), the sample was similar by SES, region, and age, but had more female respondents (68% vs 52% in the estimated population) and fewer respondents under 35 years (10% vs 19%). Height and weight data were asked from the respondents in the survey, and obesity prevalence was similar to that of the general population.

**Table 1:** Sample demographic characteristics

Sociodemographics	n (%)	Population target (%)*
Age (years)		
18–35	106 (10)	19
35–44	278 (27)	24
45–54	310 (30)	24
55–64	213 (21)	20
65+	133 (13)	14
Gender		
Female	712 (68)	52
Male	328 (32)	49
SES†		
AB	233 (22)	22
C1	466 (45)	44
C2	182 (18)	17
D	107 (10)	13
E	52 (5)	5
Region		
East	156 (15)	12
London	76 (7)	11
Midland	218 (21)	21
North	357 (34)	32
South	233 (22)	24
BMI		
Weight reported	851 (82)	–
Normal and underweight	317 (37)‡	36§
Overweight	294 (35)‡	38§
Obese	240 (28)‡	26§
Missing	189 (18)	–

* Target for a representative sample of online takeaway consumers in England, estimated from Kantar’s Worldpanel OOH purchase panel, online food delivery, 52 w/e 17 April 2022 (Kantar’s estimate).

† AB – higher and intermediate managerial, administrative, professional occupations; C1 – supervisory, clerical and junior managerial, administrative and professional; C2 – skilled manual workers; D – semi-skilled and unskilled manual workers; E – state pensioners, casual and lowest grade workers, unemployed with state benefits only.^20^

‡ % calculated from non-missing observations.

§ Based on data for English population.^32^ Authors’ own analysis of Kantar’s Worldpanel Panel Voice survey of 1040 respondents, November 2022.

BMI, body mass index; OOH, out-of-home; SES, occupational socioeconomic status.

### Frequency of takeaway use

**Figure 1:**
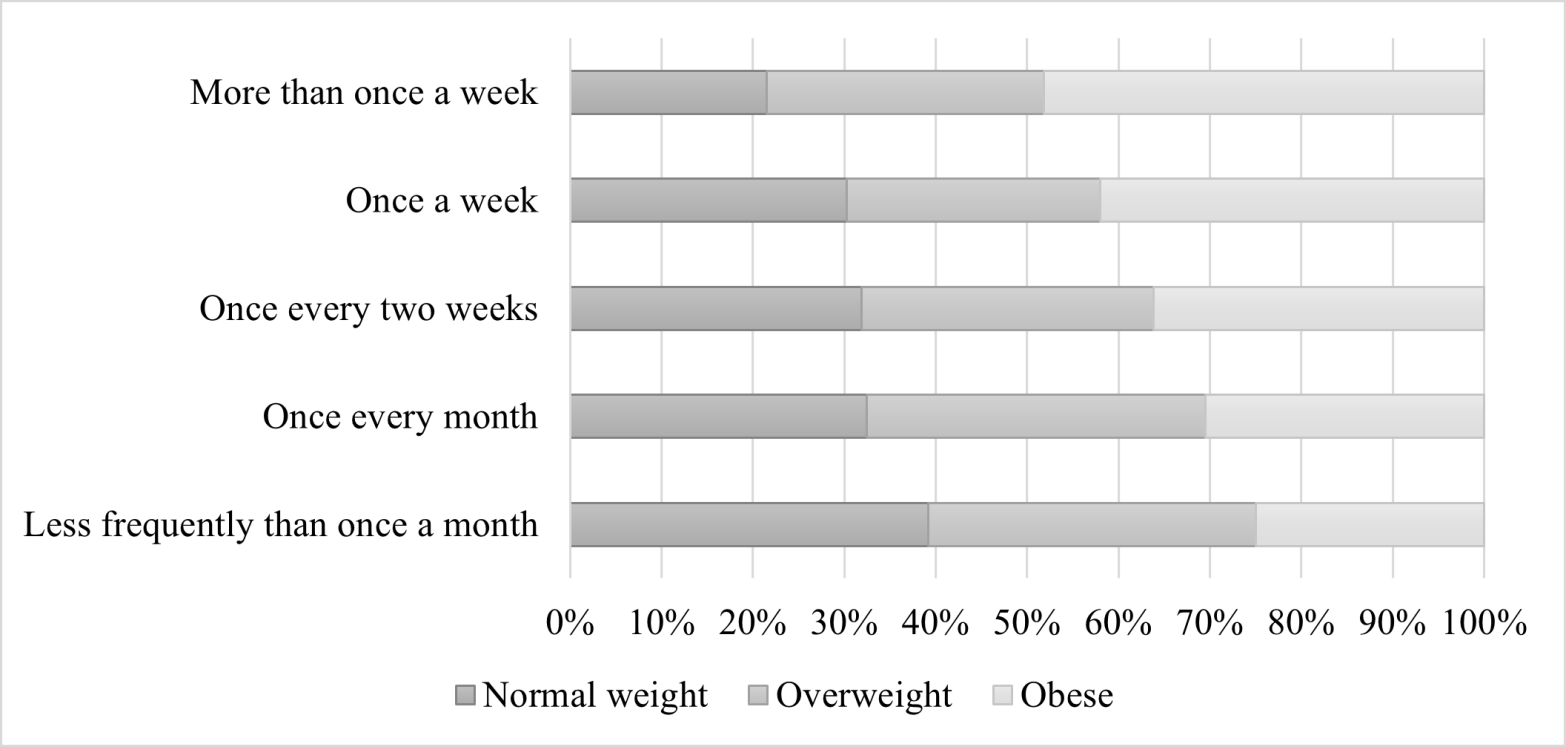
Takeaway frequency by weight status. Cross-tabulation of takeaway frequency by weight status (χ²(8) = 21.37, p=0.006). Authors' own analysis of Kantar’s Worldpanel Panel Voice survey of 1040 respondents, November 2022.

Over a quarter of the sample (27%) reported having takeaway food at least weekly. A further 41% had takeaways every 2 weeks to once a month, and 32% less than once a month (table 2). Frequency varied by age (table 3, see online supplemental table S2 for full results with confidence intervals (CIs)), with those aged 35 and older less likely to have frequent takeaways compared with under 35s (odds ratio (OR) ranged from 0.28–0.52, p<0.05). Individuals with obesity were twice as likely to have weekly takeaways (OR 2.01, p<0.001) compared with those of healthy weight or underweight (figure 1, table 3, see online supplemental table S2 for full results with CIs).

**Table 2:** Descriptive summary of outcomes

	n (%) All response categories	n (%) As coded for analysis
Frequency of takeaway consumption		
More than once a week	65 (6.2%)	280 (26.9%)
Once a week	215 (20.7%)	
Once every 2 weeks	201 (19.3%)	760 (73.1%)
Once every month	233 (22.4%)	
Less frequently than once a month	326 (31.3%)	
Recommended calorie content		
Mean (SE)	747 (11.5) kcal	
Above 600	545 (52.4%)	
600	155 (14.9%)	
Below 600	340 (32.7%)	
Know about labelling policy		
Yes	654 (62.9%)	
No	386 (37.1%)	
Noticed labelling last takeaway order		
Yes	235 (22.6%)	
No	805 (77.4%)	
Among those who noticed, how was choice affected (n=235)		
I ordered items with fewer calories than I would have had I not known calorie content (food)	62 (26.4%)	
I ordered items with more calories than I would have had I not known calorie content (food)	6 (2.6%)	
It did not influence my choice (food)	167 (71.1%)	
I ordered items with fewer calories than I would have had I not known calorie content (drink)	23 (9.8%)	
I ordered items with more calories than I would have had I not known calorie content (drink)	5 (2.1%)	
It did not influence my choice (drink)	150 (63.4%)	
Importance of drivers of choice of takeaway meal on a scale 1–5	Mean (SD) score	n (%) scoring 4–5
Healthiness	2.8 (1.1)	228 (21.9%)
Price	4.1 (0.93)	771 (74.1%)
Taste	4.7 (0.60)	988 (95.0%)
Delivery time	3.7 (1.1)	633 (60.9%)
Portion size	3.7 (0.94)	607 (58.3%)
Produced/transported with a low carbon footprint	2.5 (1.1)	163 (15.7%)
Drivers of choice of home cooked meal on a scale 1–5	Mean (SD) score	n (%) scoring 4–5
Healthiness	3.6 (0.93)	581 (55.9%)
Price	3.9 (0.96)	711 (68.4%)
Taste	4.6 (0.63)	978 (94.0%)
Preparation time	3.8 (1.0)	651 (62.6%)
Ease of preparation	3.8 (0.99)	676 (65.0%)
Portion size	3.8 (0.88)	654 (62.9%)
Produced/transported with a low carbon footprint	2.6 (1.1)	181 (17.4%)
Support for policies to encourage healthier choices of takeaway foods on a scale 1–5	Mean (SD) score	n (%) scoring 4–5
Higher prices on less healthy foods and drinks	2.8 (1.2)	35 (30.3%)
Smaller portions	2.9 (1.1)	311 (29.9%)
Information on the amount of exercise needed to spend the number of kcal eaten	2.9 (1.2)	318 (30.6%)
Traffic-light style labels showing the calorie levels of food	3.5 (1.1)	522 (50.2%)
More healthier alternatives on the menus	3.5 (1.0)	552 (53.1%)

Note: authors’ own analysis of Kantar’s Worldpanel Panel Voice survey of 1040 respondents, November 2022.

**Table 3:** Association between frequency of takeaways, knowledge and use of calorie labels and sociodemographic characteristics

	Frequency of takeaways (1- weekly or more often, 0 less frequently) OR (SE)	Knowledge of recommended energy content for a meal (1 >600 cal, 2 – 600 kcal, 3 <600 kcal)† OR (SE)	Knowledge about calorie labelling policy in OOH settings (1 yes, 0 no) OR (SE)	Noticed kcal labels last time ordered takeaway (1 yes, 0 no) OR (SE)	(If noticed kcal labels in previous takeaway) ordered fewer calories from food than without knowing energy content (1 yes, 0 no) OR (SE)
SES group (base AB)					
C1	1.01 (0.21)	0.92 (0.16)	0.63 (0.12)*	1.29 (0.28)	0.97 (0.43)
C2	1.34 (0.33)	1.16 (0.24)	0.56 (0.13)*	0.80 (0.23)	0.85 (0.52)
D	1.08 (0.33)	1.56 (0.39)	0.61 (0.17)	0.86 (0.29)	1.52 (1.08)
E	1.02 (0.43)	1.25 (0.46)	0.59 (0.22)	1.10 (0.49)	0.24 (0.29)
Sex (base male)					
Female	0.86 (0.15)	2.06 (0.32)**	1.25 (0.20)	1.07 (0.20)	1.54 (0.63)
Age group (base under 35 years)					
35–44	0.41 (0.12)**	1.40 (0.36)	1.00 (0.28)	1.26 (0.41)	1.45 (1.00)
45–54	0.52 (0.15)*	1.27 (0.33)	0.96 (0.27)	1.04 (0.34)	1.87 (1.28)
55–64	0.41 (0.12)**	1.16 (0.32)	0.82 (0.24)	0.92 (0.33)	0.59 (0.46)
65+	0.28 (0.10)**	1.56 (0.46)	0.76 (0.24)	0.87 (0.33)	0.45 (0.41)
Region (base London)					
East	0.66 (0.24)	0.61 (0.20)	2.51 (0.83)*	0.77 (0.30)	1.36 (1.08)
Midlands	0.74 (0.25)	0.76 (0.23)	2.34 (0.72)*	1.39 (0.50)	0.91 (0.64)
North	0.92 (0.30)	0.94 (0.27)	1.64 (0.48)	0.64 (0.23)	0.73 (0.53)
South	0.88 (0.30)	0.59 (0.18)	2.01 (0.62)*	0.94 (0.34)	0.46 (0.35)
BMI category (base underweight/normal weight)					
Overweight	1.07 (0.22)	0.90 (0.15)	1.00 (0.18)	1.46 (0.30)	1.07 (0.47)
Obese	2.01 (0.40)**	1.09 (0.19)	1.06 (0.20)	1.32 (0.29)	1.38 (0.65)
Constant	0.76 (0.31)		1.19 (0.46)	0.21 (0.10)**	0.28 (0.26)
Number of observations	851	834	851	851	184
Pseudo R2	0.04	0.03	0.02	0.03	0.07
Probability >χ^2^	<0.01	<0.01	0.05	0.08	0.43

For outcomes reported in this table BMI was deemed missing at random (see further details in methods). Detailed results including CIs are available in online supplemental table S2. Authors' own analysis of Kantar’s Worldpanel Panel Voice survey of 1040 respondents, November 2022.

*p<0.05; **p<0.01.

Multivariate logistic (†ordered logistic) regression. Excludes n=189 respondents for whom weight or height data were missing and BMI could not be calculated.

BMI, body mass index; Obs, Number of observations; PROB, Probability; SES, occupational socioeconomic status.

### Knowledge of recommended calorie content of a meal

Respondents’ average estimate of the recommended calorie content for a meal was 747 kcal, about 25% higher than recommended by the guidelines (600 kcal).^18^ A third (33%) thought it was less than 600 kcal, 15% correctly identified 600 kcal, and 53% thought it was over 600 kcal (table 2). Women had higher odds (OR 2.06, p<0.001) of reporting the correct (600 kcal) or lower than the recommended (<600 kcal) meal calorie content (table 3, see online supplemental table S2 for full results with CIs).

### Knowledge of calorie labelling policy

Almost two thirds (63%) of respondents were aware of the requirement for OOH food outlets to display calorie content (table 2). Middle SES respondents (C1 and C2) had lower odds of awareness compared with highest SES (AB) respondents (OR 0.56–0.63, p<0.05) (table 3, see online supplemental table S2) for full results with CIs). Except in the North of England, respondents in all regions outside London were approximately twice as likely to be aware of the policy (OR 2.01–2.51, p<0.05).

### Noticing calorie labels and their perceived impact on last meal ordered online

The majority (77%) of respondents did not notice any calorie information during their most recent online takeaway purchase, and there were no significant sociodemographic differences in noticing calorie labels (table 2). Of the 23% who noticed calorie labels (n=235), 71% said it did not affect their food choices and 63% reported no impact on their drink choices. However, 26% and 10% of respondents who noticed calorie labels reported ordering fewer calories from food and drinks, respectively, while 2–3% of respondents reported ordering more calories. This did not vary by sociodemographic characteristics (table 3, see online supplemental table S2) for full results with CIs).

### Drivers of choice

**Figure 2:**
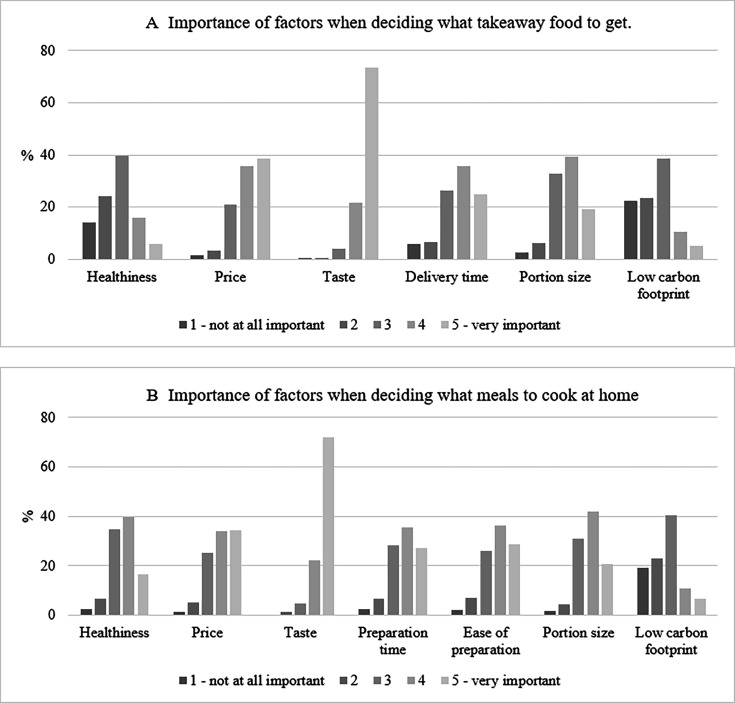
Importance of drivers of choice for takeaways (A) compared with home-cooked meals (B). Respondents were asked to rank the score on a scale from 1 (not at all important) to 5 (very important) ‘how important the following factors are to you when deciding what takeaway food to get?’. The figures represent % of respondents for each score and each driver. Authors' own analysis of Kantar’s Worldpanel Panel Voice survey of 1040 respondents, November 2022.

Figure 2 shows respondents’ ranking of takeaway and home-cooked meal drivers. For takeaways, taste was the most important factor, followed by price, delivery time and portion size. For home-cooked meals, taste and price were also two leading drivers, followed by portion size, preparation time and ease. Healthiness was more important for home-cooked meals as 56% respondents scored healthiness as (very) important compared with 22% for takeaways. Low carbon footprint was the least relevant driver for both meal types.

Women were more likely than men to consider healthiness (very) important for takeaway (OR 1.51, p=0.031) as were those aged 65 or older (OR 4.11, p<0.001) (table 4, see online supplemental table S3 for full results with CIs). For home-cooked meals, lower SES respondents were less likely to prioritise healthiness compared with higher SES (OR 0.34–0.60, p<0.05) as were those in two of the regions (North and South) outside London (OR 0.43–0.51, p<0.05) . Women were also more likely to rate healthiness as important for home-cooked meals than men (OR 2.06, p<0.001), as did all age groups compared with those under 35 (OR 1.97–3.05, p<0.05). Those living with obesity (but not overweight) were less likely to rank healthiness as an important driver (OR 0.5, p<0.001) for home-cooked meal choice. However, findings for drivers of home meal choice should be interpreted with caution as BMI was not missing at random for this outcome (ie, ranking healthiness important was associated with lower odds of BMI missing (OR 0.65, p=0.011).

**Table 4:** Association between takeaway and home meal drivers of choice and sociodemographic characteristics

	Drivers of takeaway choice: healthiness (1, score 4–5; 0, score 1–3)	Drivers of home meal choice: healthiness (1, score 4–5; 0, score 1–3)†	Drivers of takeaway choice: taste (1, score 4–5; 0, score 1–3)	Drivers of home meal choice: taste (1, score 4–5; 0, score 1–3)	Drivers of takeaway choice: low carbon footprint (1, score 4–5; 0, score 1–3)	Drivers of home meal choice: low carbon footprint (1, score 4–5; 0, score 1–3)
	OR (SE)	OR (SE)	OR (SE)	OR (SE)	OR (SE)	OR (SE)
SES group (base AB)						
C1	1.1 (0.24)	0.78 (0.15)	0.95 (0.39)	1.53 (0.61)	1.22 (0.31)	1.06 (0.25)
C2	1.05 (0.28)	0.6 (0.14)*	1.11 (0.58)	1.35 (0.64)	1.11 (0.34)	0.66 (0.2)
D	1.16 (0.37)	0.43 (0.12)**	1.53 (1.06)	0.91 (0.49)	1.01 (0.38)	0.73 (0.26)
E	0.71 (0.33)	0.34 (0.13)*	0.36 (0.24)	0.26 (0.16)*	1.04 (0.53)	0.34 (0.22)
Sex (base male)						
Female	1.51 (0.29)*	2.06 (0.33)**	2.45 (0.83)*	1.79 (0.6)	1.54 (0.34)*	1.55 (0.34)*
Age group (base under 35 years)						
35–44	0.95 (0.34)	2.6 (0.73)**	3.57 (1.68)*	2.52 (1.06)*	0.88 (0.36)	0.72 (0.25)
45–54	1.31 (0.45)	1.97 (0.55)*	5.19 (2.54)**	3.26 (1.44)*	1.42 (0.56)	0.6 (0.22)
55–64	1.89 (0.67)*	2.66 (0.78)**	6.05 (3.4)**	8.87 (5.47)**	2.3 (0.92)*	1.05 (0.38)
65+	4.11 (1.48)**	3.05 (0.97)**	5.63 (3.38)**	32.21 (34.65)**	2.56 (1.07)*	1.7 (0.63)
Region (base London)						
East	1.04 (0.39)	0.58 (0.2)	1.8 (1.21)	0.81 (0.55)	0.48 (0.19)	0.62 (0.25)
Midlands	0.91 (0.33)	0.56 (0.19)	1.31 (0.76)	1.04 (0.66)	0.65 (0.24)	0.63 (0.24)
North	0.82 (0.28)	0.43 (0.14)*	1.38 (0.78)	0.7 (0.43)	0.38 (0.14)*	0.61 (0.22)
South	0.69 (0.25)	0.51 (0.17)*	1.71 (1.05)	1.14 (0.75)	0.51 (0.19)	0.79 (0.3)
BMI category (base underweight/normal weight)						
Overweight	1 (0.2)	0.84 (0.15)	0.72 (0.28)	1.35 (0.49)	0.93 (0.21)	1.04 (0.23)
Obesity	0.68 (0.15)	0.5 (0.09)**	1.19 (0.54)	2.39 (1.06)*	0.7 (0.18)	0.65 (0.17)
Constant	0.18 (0.08)**	1.31 (0.52)	2.29 (1.49)	2.55 (1.69)	0.17 (0.09)**	0.3 (0.14)*
Number of observations	851	851	851	851	851	851
Pseudo R2	0.05	0.06	0.07	0.11	0.04	0.04
Probability>χ^2^	<0.01	<0.01	0.05	<0.01	0.02	0.01

Multivariate logistic regression. Selected outcomes only. Results for remaining outcomes are in table S4online supplemental table S4.table S3

Excludes n=189 respondents for whom weight or height data were missing and BMI could not be calculated.

Detailed results including CIs are available in online supplemental table S3. Authors' own analysis of Kantar’s Worldpanel Panel Voice survey of 1040 respondents, November 2022.

*p<0.05; **p<0.01.

†BMI was not missing at random (those who scored 4–5 were less likely to have BMI missing).

BMI, body mass index; Obs, Number of observations; PROB, Probability; SES, occupational socioeconomic status.

The odds of ranking taste as an important driver for both home and takeaway meals increased in each age group compared with the base (under 35 year olds) (OR 2.52–32.21, p<0.05). For home meals taste was also more likely to be ranked as an important driver among those living with obesity (OR 2.39, p=0.05). Women were more likely (OR 1.54–1.55, p<0.05) to rank low carbon footprint as an important driver for both home and takeaway meals compared with men. Remaining drivers exhibited fewer patterns by sociodemographic characteristics (see online supplemental table S4 for results).

### Perceptions of policies to encourage healthier takeaway choices

**Figure 3:**
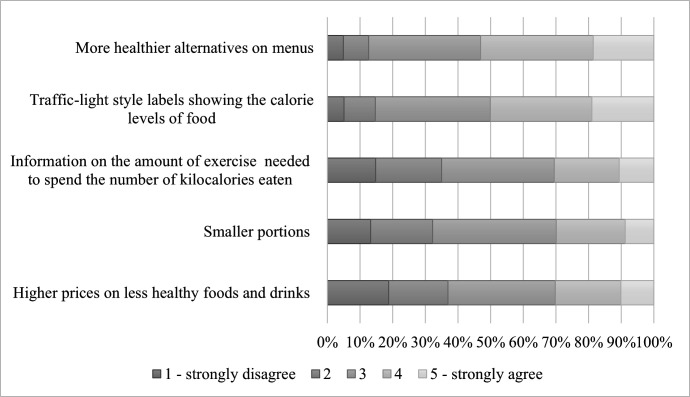
Perceptions of policies to encourage healthier takeaway choices. Respondents were asked on a scale from 1 (strongly disagree) to 5 (strongly agree), ‘to what extent do you believe that the following would encourage people to make healthier takeaway food choices?’. The figures represent % of respondents for each score for each policy. Authors' own analysis of Kantar’s Worldpanel Panel Voice survey of 1040 respondents, November 2022.

Figure 3 shows that around 50% of respondents agreed or strongly agreed that traffic-light labels and more healthy alternatives would encourage healthier choices, while around 30% supported higher prices, exercise-based calorie labels, and smaller portions (table 2). Women were more likely to support smaller portions (OR 2.01, p<0.001), while lower SES respondents (class C2 and D) were less likely to support this (OR 0.53–0.58, p<0.05) or healthier menu options (class D, OR 0.47, p=0.044) (online supplemental table S4). There was no difference by sociodemographic characteristics in support for labelling policies (online supplemental table S4).

### Thematic analysis

The thematic analysis of the valid responses (n=298) revealed three key themes (see online supplemental material S5 for additional quotes). First, some respondents saw takeaways as occasional treats and unlikely to be influenced by policy. They acknowledged takeaways as an unhealthy option to be consumed in moderation, and therefore calorie labelling or any similar policy was seen as having little or no influence on choice.

‘…people consider takeaways as treats, and calorie content probably isn’t on the priority list’ (Kantar’s Worldpanel Panel Voice survey, November 2022)

The second theme highlighted the need for policies targeting the broader food environment. Respondents suggested a wider range of portion sizes, lowering the price of healthier food options through discounts and loyalty schemes and improving the variety and quality of healthier alternatives. Some noted that higher prices on less healthy alternatives might discourage purchases altogether rather than prompting a switch to healthier alternatives.

Buy one get one free on healthier options but NOT on the unhealthy option (Kantar’s Worldpanel Panel Voice survey, November 2022)

A third theme emphasised the need for policies focused on health education and improved information. Suggestions included public health campaigns, nutrition and cooking education in schools, and clearer ingredients and calorie information. Some participants supported more detailed labelling, such as a traffic light system or ‘exercise-equivalent’ calorie labels or health warnings similar to those on tobacco and alcohol.

Better education in schools on ‘realistic food preparation’' as well as further adult education on healthy cooking on a time-and-money saving budget (Kantar’s Worldpanel Panel Voice survey, November 2022)

## Discussion

### Key findings

Over a quarter (27%) of respondents had takeaways weekly or more often, with individuals living with obesity twice as likely to have weekly or more frequent takeaways compared with those with a healthy weight or underweight. Frequent takeaways were also more common among younger adults, with those under 35 more than twice as likely to order weekly or more often compared with older age groups. These are slightly higher estimates compared with the previous literature, which are based on population rather than takeaway consumers only. For example, a study using 2016–2017 data reported that 14% (high-SES) to 21% (low-SES) purchased takeaways and fast food weekly or more often.^22^ In 2008–12, weekly or more frequent takeaway consumption was recorded by 21% of adults in the NDNS data.^4^

A minority of respondents (15%) correctly identified 600 kcal as the recommended energy content for a meal, and more than half overestimated it. Women were twice as likely as men to know the correct recommended value or estimate a lower value. Awareness of the calorie labelling regulation was relatively high (63%) but more likely among the highest SES group. However, when reflecting on their previous online takeaway order, only 23% reported noticing calorie labels. Among those who noticed, only 26% reported ordering fewer calories from food and 10% reported ordering fewer calories from drinks. We found no sociodemographic associations with ordering fewer calories, but as this applied only to a small share of the sample, we may have lacked sufficient power to detect these.

Our findings concur with a recent study in England, where of the 19% of respondents who noticed calorie labels in physical stores, only 22% reported using them.^10^ Some prior research has also found that calorie label use is more likely among women, younger people and higher educated or higher income earners, often described as being more health conscious.^23 24^ A study of UK consumers’ use of nutrition labels (although in supermarkets) found the sociodemographic effects were entirely mediated by interest in healthy eating.^25^ Similarly, Berry *et al*, who divided consumers into health, taste and quantity value-oriented consumers, found that calorie labelling was an effective measure only for those who were health-oriented.^15^ For taste and quantity value-oriented consumers, labels may lead to an increase in calories purchased.^15^ We found that 2–3% of those who noticed labels reported ordering more calories from food and drink than they would have without this information.

Taste and price were the most important drivers in takeaway decisions, while healthiness and low carbon footprint were ranked as least important. Price and taste were also the two most important drivers for home-cooked meals. However, healthiness was significantly more important for home-cooked meals than takeaways, reinforcing the view of takeaways as a ‘treat’—a theme that also emerged from the thematic analysis. Another qualitative study with 22 online food delivery service consumers reported similar themes to those reported here, indicating that consumers were price-sensitive and did not use takeaways to purchase healthy food.^26^ Additionally, these participants reported that takeaway food was not what they would typically cook at home.^26^ Dunn *et al* suggested that taste, satisfaction and convenience often outweigh long-term health concerns in fast-food choices.^27^

Although sustainability within the food system is widely discussed, low carbon footprint was ranked as the least important driver of takeaway or home-prepared meals. This aligns with another survey which found that while most respondents were highly (27%) or somewhat (49%) concerned about sustainable food production, only 5% considered eco-friendliness the most important criterion when choosing food.^28^ To encourage healthier takeaways, over half of the respondents supported expanding healthier menu options and traffic light labels, while smaller portions and higher prices were less favoured. The thematic analysis revealed mixed views. Many respondents viewed takeaways as an occasional treat with less emphasis on health, but there were also suggestions for more policies targeting the broader food environment and nutrition education to improve the healthiness of takeaways.

### Strengths and limitations

The study had a relatively large sample of takeaway consumers in England, and findings align with previous research. However, younger (under 35) and male respondents were underrepresented, and some subgroups, such as those reporting behaviour changes due to labelling, were too small to understand further heterogeneity. BMI associations relied on self-reported data, which may be biased, and some observations were missing. While tests indicated BMI was missing at random for most outcomes, allowing complete case analysis, it did reduce sample size and therefore may have affected statistical power. Lastly, given the cross-sectional nature of the data, our findings should be considered as exploratory rather than causal.

### Implications for research and practice

Our findings highlight the complexity of efforts to improve the healthiness of takeaway foods as they are often perceived as an occasional treat where health considerations are less important compared with home-cooked meals. On the other hand, 27% of respondents reported consuming takeaway meals weekly or more often, yet over half of the overall sample overestimated the recommended calorie content of a meal. Calorie labelling was widely unnoticed and influenced only a minority of choices.

To improve the healthiness of takeaway choices, over half of respondents supported traffic-light labels, which may be more effective due to their clarity and familiarity from use on packaged goods in England.^24 29^ Exercise-based labelling, which we found to be less favoured, may be more appealing to those who are already physically active^29^ and remains less proven in encouraging healthier choices compared with calorie labelling only.^30 31^

Price, alongside taste, was a key driver for takeaway choice. While only a third supported higher prices for less healthy alternatives, there were suggestions to lower the price of healthier options. This suggests that creating a price differential (eg, either through taxes, subsidies or regulating promotions) could be important.

## Conclusion

More real-world evaluations are needed from different contexts and populations to understand motivations of takeaway consumption and the effectiveness of calorie labels in this setting, including in conjunction with other interventions. While calorie labelling may affect only a minority, it can be part of a broader strategy to address obesity and poor diets. However, its potential to improve diets without exacerbating existing health inequities remains uncertain. Ongoing efforts to understand motivations and drivers of takeaway choices are essential to complement labelling policies.

## Supplementary material

10.1136/bmjnph-2025-001268 online supplemental file 1[Aff aff1][Corresp cor2]

## Data Availability

Data may be obtained from a third party and are not publicly available.
