# Supplementary material for: Calorie labelling and other drivers of takeaway food choices
Source: BMJ Nutr Prev Health. 2025 Aug 12;8(2):e001268. doi: 10.1136/bmjnph-2025-001268 (PMC12772572; doi:10.1136/bmjnph-2025-001268)
Supplement: online supplemental file 1 [file bmjnph-8-2-s001.docx]

**Supplementary Materials**

**Table S1. Outcome variables, source survey questions and coding**

| **Outcome** | **Question** | **Response options** | **Coding for the analysis** |
| --- | --- | --- | --- |
| 1. Frequency of takeaway purchase | How often do you have takeaway food in your household? *By takeaway food, we mean food you would order from a restaurant to get delivered, collect yourself or buy when out and about / on-the-go to be eaten at home* | More than once a week; once a week; once every two weeks, once every month and less frequently than once a month | 1 - Once a week or more often  0 – less than weekly frequency |
| 2. Knowledge of the recommended calorie content | What do you think is the recommended calorie content of a main meal for lunch or dinner? | Open text (numerical value only) | 1 - >600  2 - 600  3 - <600  (values below 50 or above 4000 were excluded) |
| 3. Knowledge of labelling policy | From April 2022 some restaurants, fast-food chains and cafes in England are required by law to display the calorie information of foods and drinks on their menus. Are you aware of this requirement? | Yes/no | 1 – yes  0 – no |
| 4. Noticing labelling policy | Thinking about the last time you ordered takeaway food online, did you notice any calorie information displayed on the menu? | Yes/no | 1 – yes  0 – no |
| 5. Impact of labelling policy | How did the calorie information influence the type of takeaway foods and drinks you ordered online? | Separately for food and drink:   - I ordered items with less calories than I would have had I not known calorie content; - I ordered items with more calories than I would have had I not known calorie content; - It did not influence my choice | 1 – yes  0 – no |
| 6. Drivers of takeaway choice | Please score on a scale from 1 (Not at all important) to 5 (Very important) how important the following factors are to you when deciding what takeaway food to get. | - Healthiness - Price - Taste - Delivery time - Portion size - Produced/transported with a low carbon footprint | 1 – score 4 or 5 (important or very important)  0 – score 1-3 |
| 7. Drivers of home cooked meal choice | Please score on a scale from 1 (Not at all important) to 5 (Very important) how important the following factors are to you when deciding what meals to cook at home. | - Healthiness - Price - Taste - Preparation time - Ease of preparation - Portion size - Produced/transported with a low carbon footprint | 1 – score 4 or 5 (important or very important)  0 – score 1-3 |
| 8. Support for policies encouraging healthier takeaways | On a scale from 1 (Strongly disagree) to 5 (Strongly agree), to what extent do you believe that the following would encourage people to make healthier takeaway food choices? | - Higher prices on less healthy foods and drinks - Smaller portions - Information on the amount of exercise needed to spend the number of kilocalories eaten - Traffic-light style labels showing the calorie levels of food, such as red for very caloric foods and green for less caloric foods - More healthier alternatives on menus | 1 – score 4 or 5 (agree or strongly agree)  0 – score 1-3 |
| 9. Support for policies encouraging healthier takeaways | Are there any other policies which you feel would encourage healthier takeaway food choices? | Free text | Thematic analysis |

**Table S2. Association between frequency of takeaways, knowledge and use of calorie labels and socio-demographic characteristics (Detailed Results)**

|  | **Frequency of takeaways**  **(1- weekly or more often, 0 less frequently)** | | | | | **Knowledge of recommended energy content for a meal**  **(1 > 600cal, 2 - 600kcal,**  **3 < 600kcal)*** | | | | | **Knowledge about calorie labelling policy in OOH settings**  **(1- yes, 0-no)** | | | | | **Noticed kcal labels last time ordered takeaway**  **(1-yes, 0-no)** | | | | | **(If noticed kcal labels in previous takeaway) ordered less calories from food than without knowing energy content (1-yes, 0-no)** | | | | |
| --- | --- | --- | --- | --- | --- | --- | --- | --- | --- | --- | --- | --- | --- | --- | --- | --- | --- | --- | --- | --- | --- | --- | --- | --- | --- |
|  | OR | SE | P>z | 95% CI | | OR | SE | P>z | 95% CI | | OR | SE | P>z | 95% CI | | OR | SE | P>z | 95% CI | | OR | SE | P>z | 95% CI | |
| **SES group (base AB)** |  |  |  |  |  |  |  |  |  |  |  |  |  |  |  |  |  |  |  |  |  |  |  |  |  |
| C1 | 1.01 | 0.21 | 0.97 | 0.67 | 1.52 | 0.92 | 0.16 | 0.66 | 0.65 | 1.31 | 0.63 | 0.12 | 0.02 | 0.43 | 0.92 | 1.29 | 0.28 | 0.24 | 0.84 | 1.98 | 0.97 | 0.43 | 0.94 | 0.40 | 2.32 |
| C2 | 1.34 | 0.33 | 0.23 | 0.83 | 2.18 | 1.16 | 0.24 | 0.49 | 0.76 | 1.75 | 0.56 | 0.13 | 0.01 | 0.36 | 0.88 | 0.80 | 0.23 | 0.42 | 0.46 | 1.39 | 0.85 | 0.52 | 0.79 | 0.26 | 2.81 |
| D | 1.08 | 0.33 | 0.80 | 0.60 | 1.96 | 1.56 | 0.39 | 0.08 | 0.95 | 2.55 | 0.61 | 0.17 | 0.08 | 0.36 | 1.05 | 0.86 | 0.29 | 0.65 | 0.44 | 1.66 | 1.52 | 1.08 | 0.56 | 0.38 | 6.11 |
| E | 1.02 | 0.43 | 0.97 | 0.45 | 2.31 | 1.25 | 0.46 | 0.54 | 0.61 | 2.56 | 0.59 | 0.22 | 0.16 | 0.28 | 1.23 | 1.10 | 0.49 | 0.83 | 0.46 | 2.65 | 0.24 | 0.29 | 0.23 | 0.02 | 2.47 |
| **Sex (base male)** |  |  |  |  |  |  |  |  |  |  |  |  |  |  |  |  |  |  |  |  |  |  |  |  |  |
| Female | 0.86 | 0.15 | 0.40 | 0.61 | 1.21 | 2.06 | 0.32 | <0.01 | 1.51 | 2.81 | 1.25 | 0.20 | 0.16 | 0.92 | 1.70 | 1.07 | 0.20 | 0.71 | 0.74 | 1.55 | 1.54 | 0.63 | 0.29 | 0.69 | 3.43 |
| **Age group (base under 35 years)** |  |  |  |  |  |  |  |  |  |  |  |  |  |  |  |  |  |  |  |  |  |  |  |  |  |
| 35-44 | 0.41 | 0.12 | <0.01 | 0.24 | 0.73 | 1.40 | 0.36 | 0.19 | 0.84 | 2.31 | 1.00 | 0.28 | 0.99 | 0.58 | 1.74 | 1.26 | 0.41 | 0.48 | 0.66 | 2.40 | 1.45 | 1.00 | 0.59 | 0.37 | 5.60 |
| 45-54 | 0.52 | 0.15 | 0.02 | 0.30 | 0.91 | 1.27 | 0.33 | 0.36 | 0.76 | 2.10 | 0.96 | 0.27 | 0.88 | 0.55 | 1.66 | 1.04 | 0.34 | 0.90 | 0.54 | 1.99 | 1.87 | 1.28 | 0.36 | 0.49 | 7.15 |
| 55-64 | 0.41 | 0.12 | <0.01 | 0.23 | 0.75 | 1.16 | 0.32 | 0.59 | 0.68 | 1.99 | 0.82 | 0.24 | 0.50 | 0.46 | 1.46 | 0.92 | 0.33 | 0.82 | 0.46 | 1.85 | 0.59 | 0.46 | 0.50 | 0.13 | 2.69 |
| 65+ | 0.28 | 0.10 | <0.01 | 0.14 | 0.55 | 1.56 | 0.46 | 0.13 | 0.87 | 2.77 | 0.76 | 0.24 | 0.37 | 0.41 | 1.39 | 0.87 | 0.33 | 0.70 | 0.41 | 1.82 | 0.45 | 0.41 | 0.38 | 0.08 | 2.64 |
| **Region (base London)** |  |  |  |  |  |  |  |  |  |  |  |  |  |  |  |  |  |  |  |  |  |  |  |  |  |
| East | 0.66 | 0.24 | 0.26 | 0.32 | 1.36 | 0.61 | 0.20 | 0.12 | 0.33 | 1.14 | 2.51 | 0.83 | 0.01 | 1.32 | 4.78 | 0.77 | 0.30 | 0.51 | 0.36 | 1.66 | 1.36 | 1.08 | 0.70 | 0.28 | 6.47 |
| Midlands | 0.74 | 0.25 | 0.38 | 0.38 | 1.44 | 0.76 | 0.23 | 0.36 | 0.42 | 1.37 | 2.34 | 0.72 | 0.01 | 1.28 | 4.29 | 1.39 | 0.50 | 0.36 | 0.69 | 2.81 | 0.91 | 0.64 | 0.90 | 0.23 | 3.63 |
| North | 0.92 | 0.30 | 0.80 | 0.49 | 1.74 | 0.94 | 0.27 | 0.82 | 0.53 | 1.65 | 1.64 | 0.48 | 0.09 | 0.92 | 2.91 | 0.64 | 0.23 | 0.21 | 0.32 | 1.29 | 0.73 | 0.53 | 0.66 | 0.18 | 2.99 |
| South | 0.88 | 0.30 | 0.71 | 0.45 | 1.71 | 0.59 | 0.18 | 0.08 | 0.33 | 1.07 | 2.01 | 0.62 | 0.02 | 1.10 | 3.68 | 0.94 | 0.34 | 0.85 | 0.46 | 1.92 | 0.46 | 0.35 | 0.31 | 0.11 | 2.01 |
| **BMI category (base underweight/normal weight)** |  |  |  |  |  |  |  |  |  |  |  |  |  |  |  |  |  |  |  |  |  |  |  |  |  |
| overweight | 1.07 | 0.22 | 0.74 | 0.72 | 1.59 | 0.90 | 0.15 | 0.50 | 0.65 | 1.24 | 1.00 | 0.18 | 1.00 | 0.71 | 1.41 | 1.46 | 0.30 | 0.07 | 0.97 | 2.19 | 1.07 | 0.47 | 0.87 | 0.46 | 2.51 |
| obese | 2.01 | 0.40 | <0.01 | 1.36 | 2.98 | 1.09 | 0.19 | 0.63 | 0.77 | 1.53 | 1.06 | 0.20 | 0.76 | 0.74 | 1.52 | 1.32 | 0.29 | 0.20 | 0.86 | 2.04 | 1.38 | 0.65 | 0.49 | 0.55 | 3.46 |
| **Constant** | 0.76 | 0.31 | 0.49 | 0.34 | 1.67 |  |  |  |  |  | 1.19 | 0.46 | 0.65 | 0.56 | 2.52 | 0.21 | 0.10 | <0.01 | 0.09 | 0.52 | 0.28 | 0.26 | 0.18 | 0.04 | 1.80 |
| Obs | 851 |  |  |  |  | 834 |  |  |  |  | 851 |  |  |  |  | 851 |  |  |  |  | 184 |  |  |  |  |
| pseudo R2 | 0.04 |  |  |  |  | 0.03 |  |  |  |  | 0.02 |  |  |  |  | 0.03 |  |  |  |  | 0.07 |  |  |  |  |
| PROB>CHI2 | <0.01 |  |  |  |  | <0.01 |  |  |  |  | 0.05 |  |  |  |  | 0.08 |  |  |  |  | 0.43 |  |  |  |  |

Notes: Multivariate logistic (*ordered logistic) regression. Excludes n=189 respondents for whom weight or height data were missing and BMI could not be calculated. Authors' own analysis of Kantar’s Worldpanel Panel Voice survey of 1,040 respondents, November 2022.

|  | **Drivers of takeaway choice: healthiness**  **(1-score 4-5, 0-score 1-3)** | | | | | **Drivers of home meal choice: healthiness**  **(1-score 4-5, 0-score 1-3)*** | | | | | **Drivers of takeaway choice: taste**  **(1-score 4-5, 0-score 1-3)** | | | | | **Drivers of home meal choice: taste**  **(1-score 4-5, 0-score 1-3)** | | | | | **Drivers of takeaway choice: low carbon footprint**  **(1-score 4-5, 0-score 1-3)** | | | | | **Drivers of home meal choice: low carbon footprint**  **(1-score 4-5, 0-score 1-3)** | | | | |
| --- | --- | --- | --- | --- | --- | --- | --- | --- | --- | --- | --- | --- | --- | --- | --- | --- | --- | --- | --- | --- | --- | --- | --- | --- | --- | --- | --- | --- | --- | --- |
|  | OR | SE | P>z | 95% CI | | OR | SE | P>z | 95% CI | | OR | SE | P>z | 95% CI | | OR | SE | P>z | 95% CI | | OR | SE | P>z | 95% CI | | OR | SE | P>z | 95% CI | |
| **SES group (base AB)** |  |  |  |  |  |  |  |  |  |  |  |  |  |  |  |  |  |  |  |  |  |  |  |  |  |  |  |  |  |  |
| C1 | 1.10 | 0.24 | 0.66 | 0.71 | 1.70 | 0.78 | 0.15 | 0.20 | 0.54 | 1.14 | 0.95 | 0.39 | 0.90 | 0.42 | 2.13 | 1.53 | 0.61 | 0.29 | 0.70 | 3.32 | 1.22 | 0.31 | 0.43 | 0.74 | 2.02 | 1.06 | 0.25 | 0.80 | 0.67 | 1.68 |
| C2 | 1.05 | 0.28 | 0.85 | 0.63 | 1.77 | 0.60 | 0.14 | 0.03 | 0.38 | 0.94 | 1.11 | 0.58 | 0.84 | 0.40 | 3.09 | 1.35 | 0.64 | 0.53 | 0.53 | 3.44 | 1.11 | 0.34 | 0.74 | 0.61 | 2.03 | 0.66 | 0.20 | 0.18 | 0.36 | 1.21 |
| D | 1.16 | 0.37 | 0.65 | 0.62 | 2.15 | 0.43 | 0.12 | <0.01 | 0.25 | 0.74 | 1.53 | 1.06 | 0.54 | 0.39 | 5.98 | 0.91 | 0.49 | 0.87 | 0.32 | 2.63 | 1.01 | 0.38 | 0.97 | 0.49 | 2.11 | 0.73 | 0.26 | 0.39 | 0.36 | 1.49 |
| E | 0.71 | 0.33 | 0.46 | 0.29 | 1.75 | 0.34 | 0.13 | 0.01 | 0.16 | 0.73 | 0.36 | 0.24 | 0.12 | 0.10 | 1.31 | 0.26 | 0.16 | 0.03 | 0.08 | 0.88 | 1.04 | 0.53 | 0.94 | 0.38 | 2.81 | 0.34 | 0.22 | 0.09 | 0.09 | 1.20 |
| **Sex (base male)** |  |  |  |  |  |  |  |  |  |  |  |  |  |  |  |  |  |  |  |  |  |  |  |  |  |  |  |  |  |  |
| Female | 1.51 | 0.29 | 0.03 | 1.04 | 2.20 | 2.06 | 0.33 | <0.01 | 1.5 | 2.81 | 2.45 | 0.83 | 0.01 | 1.26 | 4.75 | 1.79 | 0.60 | 0.08 | 0.93 | 3.44 | 1.54 | 0.34 | 0.05 | 1.00 | 2.39 | 1.55 | 0.34 | 0.05 | 1.01 | 2.38 |
| **Age group (base under 35 years)** |  |  |  |  |  |  |  |  |  |  |  |  |  |  |  |  |  |  |  |  |  |  |  |  |  |  |  |  |  |  |
| 35-44 | 0.95 | 0.34 | 0.89 | 0.48 | 1.90 | 2.6 | 0.73 | <0.01 | 1.5 | 4.51 | 3.57 | 1.68 | 0.01 | 1.42 | 8.97 | 2.52 | 1.06 | 0.03 | 1.10 | 5.76 | 0.88 | 0.36 | 0.76 | 0.39 | 1.98 | 0.72 | 0.25 | 0.34 | 0.36 | 1.43 |
| 45-54 | 1.31 | 0.45 | 0.43 | 0.67 | 2.59 | 1.97 | 0.55 | 0.02 | 1.14 | 3.41 | 5.19 | 2.54 | <0.01 | 1.99 | 13.5 | 3.26 | 1.44 | 0.01 | 1.37 | 7.74 | 1.42 | 0.56 | 0.38 | 0.65 | 3.08 | 0.60 | 0.22 | 0.15 | 0.30 | 1.21 |
| 55-64 | 1.89 | 0.67 | 0.07 | 0.95 | 3.79 | 2.66 | 0.78 | <0.01 | 1.49 | 4.74 | 6.05 | 3.40 | <0.01 | 2.02 | 18.2 | 8.87 | 5.47 | <0.01 | 2.65 | 29.7 | 2.30 | 0.92 | 0.04 | 1.04 | 5.05 | 1.05 | 0.38 | 0.89 | 0.52 | 2.14 |
| 65+ | 4.11 | 1.48 | <0.01 | 2.03 | 8.32 | 3.05 | 0.97 | <0.01 | 1.64 | 5.68 | 5.63 | 3.38 | <0.01 | 1.74 | 18.3 | 32.21 | 34.65 | <0.01 | 3.91 | 265.3 | 2.56 | 1.07 | 0.03 | 1.13 | 5.80 | 1.70 | 0.63 | 0.15 | 0.83 | 3.51 |
| **Region (base London)** |  |  |  |  |  |  |  |  |  |  |  |  |  |  |  |  |  |  |  |  |  |  |  |  |  |  |  |  |  |  |
| East | 1.04 | 0.39 | 0.93 | 0.50 | 2.15 | 0.58 | 0.2 | 0.12 | 0.29 | 1.15 | 1.80 | 1.21 | 0.38 | 0.48 | 6.74 | 0.81 | 0.55 | 0.76 | 0.21 | 3.07 | 0.48 | 0.19 | 0.07 | 0.22 | 1.06 | 0.62 | 0.25 | 0.24 | 0.28 | 1.38 |
| Midlands | 0.91 | 0.33 | 0.79 | 0.45 | 1.83 | 0.56 | 0.19 | 0.09 | 0.29 | 1.08 | 1.31 | 0.76 | 0.64 | 0.42 | 4.09 | 1.04 | 0.66 | 0.95 | 0.30 | 3.58 | 0.65 | 0.24 | 0.24 | 0.32 | 1.34 | 0.63 | 0.24 | 0.23 | 0.29 | 1.33 |
| North | 0.82 | 0.28 | 0.57 | 0.42 | 1.61 | 0.43 | 0.14 | 0.01 | 0.23 | 0.82 | 1.38 | 0.78 | 0.57 | 0.46 | 4.18 | 0.70 | 0.43 | 0.56 | 0.21 | 2.30 | 0.38 | 0.14 | 0.01 | 0.19 | 0.77 | 0.61 | 0.22 | 0.18 | 0.30 | 1.26 |
| South | 0.69 | 0.25 | 0.32 | 0.34 | 1.42 | 0.51 | 0.17 | 0.05 | 0.26 | 0.99 | 1.71 | 1.05 | 0.38 | 0.51 | 5.68 | 1.14 | 0.75 | 0.84 | 0.31 | 4.17 | 0.51 | 0.19 | 0.07 | 0.24 | 1.06 | 0.79 | 0.30 | 0.54 | 0.38 | 1.66 |
| **BMI category (base underweight/normal weight)** |  |  |  |  |  |  |  |  |  |  |  |  |  |  |  |  |  |  |  |  |  |  |  |  |  |  |  |  |  |  |
| overweight | 1.00 | 0.20 | 1.00 | 0.68 | 1.48 | 0.84 | 0.15 | 0.34 | 0.59 | 1.20 | 0.72 | 0.28 | 0.40 | 0.34 | 1.54 | 1.35 | 0.49 | 0.41 | 0.66 | 2.74 | 0.93 | 0.21 | 0.75 | 0.59 | 1.46 | 1.04 | 0.23 | 0.86 | 0.67 | 1.60 |
| obese | 0.68 | 0.15 | 0.08 | 0.44 | 1.05 | 0.5 | 0.09 | <0.01 | 0.35 | 0.72 | 1.19 | 0.54 | 0.70 | 0.49 | 2.89 | 2.39 | 1.06 | 0.05 | 1.00 | 5.68 | 0.70 | 0.18 | 0.17 | 0.42 | 1.16 | 0.65 | 0.17 | 0.09 | 0.39 | 1.08 |
| **Constant** | 0.18 | 0.08 | <0.01 | 0.07 | 0.44 | 1.31 | 0.52 | 0.49 | 0.6 | 2.87 | 2.29 | 1.49 | 0.20 | 0.64 | 8.18 | 2.55 | 1.69 | 0.16 | 0.70 | 9.36 | 0.17 | 0.09 | <0.01 | 0.06 | 0.47 | 0.30 | 0.14 | 0.01 | 0.12 | 0.75 |
| Obs | 851 |  |  |  |  | 851 |  |  |  |  | 851 |  |  |  |  | 851 |  |  |  |  | 851 |  |  |  |  | 851 |  |  |  |  |
| pseudo R2 | 0.05 |  |  |  |  | 0.06 |  |  |  |  | 0.07 |  |  |  |  | 0.11 |  |  |  |  | 0.04 |  |  |  |  | 0.04 |  |  |  |  |
| PROB>CHI2 | <0.01 |  |  |  |  | <0.01 |  |  |  |  | 0.05 |  |  |  |  | <0.01 |  |  |  |  | 0.02 |  |  |  |  | 0.01 |  |  |  |  |

**Table S3. Association between takeaway and home meal drivers of choice and socio-demographic characteristics (Detailed Results)**

Notes: Multivariate logistic regression. Selected outcomes only. Results for remaining outcomes are in table S2 in Supplementary material. Excludes n=189 respondents for whom weight or height data were missing and BMI could not be calculated.* BMI was not missing at random (those who scored 4-5 were less likely to have BMI missing). Authors' own analysis of Kantar’s Worldpanel Panel Voice survey of 1,040 respondents, November 2022.

**Table S4. Association Between Outcomes and Socio-demographic Characteristics**

|  | **(If noticed kcal labels in previous takeaway) ordered less calories from drinks than without knowing energy content**  **(1-yes, 0-no)** | | | | | **Drivers of takeaway choice: price**  **(1 - score 4-5, 0 - score 1-3)** | | | | | **Drivers of home meal choice: price***  **(1 - score 4-5, 0 - score 1-3)** | | | | | **Drivers of home meal choice: preparation time (1 - score 4-5, 0 - score 1-3)** | | | | | **Drivers of home meal choice: ease of preparation (1 - score 4-5, 0 - score 1-3)** | | | | |
| --- | --- | --- | --- | --- | --- | --- | --- | --- | --- | --- | --- | --- | --- | --- | --- | --- | --- | --- | --- | --- | --- | --- | --- | --- | --- |
|  | **OR** | **SE** | **P>z** | **95% CI** | | **OR** | **SE** | **P>z** | **95% CI** | | **OR** | **SE** | **P>z** | **95% CI** | | **OR** | **SE** | **P>z** | **95% CI** | | **OR** | **SE** | **P>z** | **95% CI** | |
| **SES group (base AB)** |  |  |  |  |  |  |  |  |  |  |  |  |  |  |  |  |  |  |  |  |  |  |  |  |  |
| C1 | 2.41 | 2.01 | 0.29 | 0.47 | 12.31 | 0.99 | 0.20 | 0.96 | 0.67 | 1.47 | 1.05 | 0.2 | 0.79 | 0.72 | 1.54 | 1.26 | 0.23 | 0.21 | 0.88 | 1.81 | 0.94 | 0.18 | 0.75 | 0.65 | 1.37 |
| C2 | 0.51 | 0.67 | 0.61 | 0.04 | 6.68 | 1.34 | 0.34 | 0.25 | 0.81 | 2.21 | 1.1 | 0.26 | 0.69 | 0.69 | 1.75 | 1.08 | 0.24 | 0.73 | 0.70 | 1.67 | 0.82 | 0.19 | 0.38 | 0.52 | 1.28 |
| D | 5.10 | 5.55 | 0.14 | 0.60 | 43.09 | 1.97 | 0.67 | 0.05 | 1.01 | 3.83 | 1.55 | 0.47 | 0.15 | 0.86 | 2.81 | 2.50 | 0.75 | <0.00 | 1.39 | 4.51 | 1.11 | 0.31 | 0.72 | 0.64 | 1.93 |
| E | 2.52 | 3.53 | 0.51 | 0.16 | 39.10 | 0.91 | 0.37 | 0.81 | 0.41 | 2.00 | 1.5 | 0.63 | 0.34 | 0.66 | 3.42 | 1.00 | 0.37 | 0.99 | 0.49 | 2.06 | 0.86 | 0.32 | 0.68 | 0.41 | 1.79 |
| **Sex (base male)** |  |  |  |  |  |  |  |  |  |  |  |  |  |  |  |  |  |  |  |  |  |  |  |  |  |
| Female | 1.71 | 1.11 | 0.41 | 0.48 | 6.11 | 1.22 | 0.21 | 0.26 | 0.87 | 1.71 | 1.28 | 0.21 | 0.14 | 0.93 | 1.77 | 1.19 | 0.19 | 0.27 | 0.87 | 1.62 | 1.18 | 0.19 | 0.30 | 0.86 | 1.61 |
| **Age group (base under 35 years)** |  |  |  |  |  |  |  |  |  |  |  |  |  |  |  |  |  |  |  |  |  |  |  |  |  |
| 35-44 | 0.50 | 0.49 | 0.47 | 0.07 | 3.39 | 2.11 | 0.63 | 0.01 | 1.18 | 3.78 | 1.88 | 0.54 | 0.03 | 1.07 | 3.29 | 2.13 | 0.59 | 0.01 | 1.23 | 3.68 | 1.56 | 0.44 | 0.12 | 0.90 | 2.71 |
| 45-54 | 1.42 | 1.29 | 0.70 | 0.24 | 8.40 | 1.63 | 0.47 | 0.10 | 0.92 | 2.88 | 1.54 | 0.44 | 0.13 | 0.89 | 2.68 | 1.81 | 0.50 | 0.03 | 1.06 | 3.11 | 1.29 | 0.36 | 0.36 | 0.75 | 2.23 |
| 55-64 | 0.17 | 0.23 | 0.18 | 0.01 | 2.30 | 1.94 | 0.61 | 0.03 | 1.05 | 3.59 | 1.47 | 0.44 | 0.19 | 0.82 | 2.64 | 1.50 | 0.43 | 0.16 | 0.85 | 2.64 | 1.21 | 0.35 | 0.52 | 0.68 | 2.14 |
| 65+ | 0.50 | 0.57 | 0.54 | 0.05 | 4.74 | 1.69 | 0.56 | 0.11 | 0.89 | 3.24 | 1.37 | 0.43 | 0.32 | 0.74 | 2.54 | 1.11 | 0.34 | 0.72 | 0.61 | 2.02 | 0.95 | 0.29 | 0.87 | 0.52 | 1.73 |
| **Region (base London)** |  |  |  |  |  |  |  |  |  |  |  |  |  |  |  |  |  |  |  |  |  |  |  |  |  |
| East | 1.32 | 1.44 | 0.80 | 0.15 | 11.25 | 0.89 | 0.32 | 0.76 | 0.44 | 1.81 | 0.79 | 0.28 | 0.5 | 0.4 | 1.57 | 1.48 | 0.49 | 0.24 | 0.77 | 2.83 | 0.69 | 0.24 | 0.29 | 0.35 | 1.37 |
| Midlands | 0.28 | 0.28 | 0.21 | 0.04 | 2.03 | 1.14 | 0.39 | 0.70 | 0.58 | 2.24 | 0.86 | 0.29 | 0.65 | 0.45 | 1.65 | 1.25 | 0.39 | 0.48 | 0.68 | 2.30 | 0.84 | 0.28 | 0.60 | 0.44 | 1.61 |
| North | 0.41 | 0.41 | 0.38 | 0.06 | 2.93 | 1.12 | 0.37 | 0.73 | 0.58 | 2.15 | 0.85 | 0.27 | 0.61 | 0.45 | 1.59 | 1.18 | 0.36 | 0.57 | 0.66 | 2.13 | 0.65 | 0.21 | 0.17 | 0.35 | 1.21 |
| South | 0.51 | 0.52 | 0.51 | 0.07 | 3.66 | 1.01 | 0.35 | 0.97 | 0.52 | 1.99 | 1.02 | 0.34 | 0.96 | 0.52 | 1.97 | 1.23 | 0.38 | 0.52 | 0.66 | 2.27 | 0.76 | 0.25 | 0.40 | 0.39 | 1.45 |
| **BMI category (base underweight/normal weight)** |  |  |  |  |  |  |  |  |  |  |  |  |  |  |  |  |  |  |  |  |  |  |  |  |  |
| overweight | 1.45 | 1.03 | 0.60 | 0.36 | 5.82 | 0.89 | 0.17 | 0.55 | 0.61 | 1.30 | 1.01 | 0.18 | 0.98 | 0.7 | 1.44 | 0.83 | 0.15 | 0.30 | 0.59 | 1.18 | 0.81 | 0.14 | 0.23 | 0.57 | 1.14 |
| obese | 0.93 | 0.71 | 0.93 | 0.21 | 4.16 | 0.97 | 0.20 | 0.90 | 0.65 | 1.47 | 1.25 | 0.25 | 0.27 | 0.84 | 1.84 | 0.77 | 0.14 | 0.17 | 0.54 | 1.12 | 0.84 | 0.16 | 0.35 | 0.58 | 1.21 |
| **Constant** | 0.09 | 0.12 | 0.08 | 0.01 | 1.34 | 1.39 | 0.57 | 0.42 | 0.62 | 3.12 | 1.3 | 0.52 | 0.51 | 0.59 | 2.86 | 0.73 | 0.28 | 0.42 | 0.35 | 1.55 | 2.20 | 0.88 | 0.05 | 1.01 | 4.81 |
| Obs | 184 |  |  |  |  | 851 |  |  |  |  | 851 |  |  |  |  | 851 |  |  |  |  | 851 |  |  |  |  |
| pseudo R2 | 0.12 |  |  |  |  | 0.02 |  |  |  |  | 0.01 |  |  |  |  | 0.03 |  |  |  |  | 0.01 |  |  |  |  |
| PROB>CHI2 | 0.48 |  |  |  |  | 0.42 |  |  |  |  | 0.60 |  |  |  |  | 0.02 |  |  |  |  | 0.53 |  |  |  |  |

Notes: Multivariate logistic regression. Excludes n=189 respondents for whom weight or height data were missing and BMI could not be calculated.* BMI was not missing at random (those who scored 4-5 were less likely to have BMI missing). Authors' own analysis of Kantar’s Worldpanel Panel Voice survey of 1,040 respondents, November 2022

**Table S4 cont.**

|  | **Drivers of takeaway choice: portion size**  **(1 - score 4-5, 0 - score 1-3)** | | | | | **Drivers of home meal choice: portion size**  **(1 - score 4-5, 0 - score 1-3)** | | | | | **Drivers of takeaway choice: delivery time**  **(1 - score 4-5, 0 - score 1-3)** | | | | | **Support for policies:**  **higher prices**  **(1 - score 4-5, 0 - score 1-3)** | | | | | **Support for policies:**  **smaller portions**  **(1 - score 4-5, 0 - score 1-3)** | | | | |
| --- | --- | --- | --- | --- | --- | --- | --- | --- | --- | --- | --- | --- | --- | --- | --- | --- | --- | --- | --- | --- | --- | --- | --- | --- | --- |
|  | OR | SE | P>z | 95% CI | | OR | SE | P>z | 95% CI | | OR | SE | P>z | 95% CI | | OR | SE | P>z | 95% CI | | OR | SE | P>z | 95% CI | |
| **SES group (base AB)** |  |  |  |  |  |  |  |  |  |  |  |  |  |  |  |  |  |  |  |  |  |  |  |  |  |
| C1 | 1.10 | 0.20 | 0.61 | 0.77 | 1.57 | 0.85 | 0.16 | 0.38 | 0.59 | 1.23 | 1.23 | 0.22 | 0.26 | 0.86 | 1.76 | 0.75 | 0.14 | 0.13 | 0.52 | 1.09 | 0.76 | 0.15 | 0.15 | 0.52 | 1.10 |
| C2 | 0.96 | 0.21 | 0.85 | 0.62 | 1.48 | 0.94 | 0.21 | 0.80 | 0.60 | 1.47 | 1.25 | 0.28 | 0.32 | 0.81 | 1.93 | 0.50 | 0.12 | 0.01 | 0.31 | 0.81 | 0.58 | 0.14 | 0.02 | 0.36 | 0.93 |
| D | 1.35 | 0.37 | 0.27 | 0.79 | 2.32 | 1.35 | 0.39 | 0.29 | 0.77 | 2.37 | 1.90 | 0.54 | 0.02 | 1.09 | 3.32 | 0.70 | 0.20 | 0.21 | 0.40 | 1.22 | 0.53 | 0.16 | 0.03 | 0.29 | 0.95 |
| E | 1.21 | 0.45 | 0.62 | 0.58 | 2.50 | 0.55 | 0.20 | 0.10 | 0.27 | 1.13 | 0.85 | 0.31 | 0.66 | 0.42 | 1.74 | 0.59 | 0.24 | 0.19 | 0.26 | 1.30 | 0.61 | 0.25 | 0.23 | 0.27 | 1.36 |
| **Sex (base male)** |  |  |  |  |  |  |  |  |  |  |  |  |  |  |  |  |  |  |  |  |  |  |  |  |  |
| Female | 0.72 | 0.11 | 0.04 | 0.53 | 0.98 | 1.06 | 0.17 | 0.71 | 0.78 | 1.44 | 1.36 | 0.21 | 0.05 | 1.00 | 1.85 | 0.82 | 0.13 | 0.23 | 0.60 | 1.13 | 2.01 | 0.36 | <0.01 | 1.41 | 2.85 |
| **Age group (base under 35 years)** |  |  |  |  |  |  |  |  |  |  |  |  |  |  |  |  |  |  |  |  |  |  |  |  |  |
| 35-44 | 2.10 | 0.57 | 0.01 | 1.24 | 3.58 | 1.56 | 0.43 | 0.10 | 0.91 | 2.66 | 1.48 | 0.40 | 0.15 | 0.87 | 2.51 | 1.01 | 0.30 | 0.96 | 0.57 | 1.81 | 0.89 | 0.27 | 0.71 | 0.49 | 1.63 |
| 45-54 | 2.24 | 0.61 | <0.01 | 1.31 | 3.82 | 1.61 | 0.44 | 0.08 | 0.94 | 2.75 | 1.86 | 0.51 | 0.02 | 1.09 | 3.18 | 1.06 | 0.31 | 0.85 | 0.59 | 1.87 | 1.16 | 0.35 | 0.62 | 0.64 | 2.10 |
| 55-64 | 1.68 | 0.48 | 0.07 | 0.97 | 2.94 | 1.81 | 0.52 | 0.04 | 1.03 | 3.20 | 2.08 | 0.60 | 0.01 | 1.18 | 3.66 | 0.96 | 0.30 | 0.90 | 0.52 | 1.76 | 1.49 | 0.47 | 0.21 | 0.80 | 2.76 |
| 65+ | 1.72 | 0.52 | 0.07 | 0.95 | 3.11 | 1.68 | 0.52 | 0.09 | 0.92 | 3.07 | 1.60 | 0.49 | 0.12 | 0.88 | 2.90 | 0.82 | 0.27 | 0.56 | 0.43 | 1.58 | 1.54 | 0.51 | 0.19 | 0.81 | 2.96 |
| **Region (base London)** |  |  |  |  |  |  |  |  |  |  |  |  |  |  |  |  |  |  |  |  |  |  |  |  |  |
| East | 0.78 | 0.26 | 0.46 | 0.41 | 1.50 | 0.91 | 0.30 | 0.78 | 0.48 | 1.74 | 0.97 | 0.32 | 0.93 | 0.51 | 1.86 | 0.73 | 0.25 | 0.34 | 0.37 | 1.41 | 1.68 | 0.65 | 0.18 | 0.79 | 3.58 |
| Midlands | 0.75 | 0.24 | 0.36 | 0.40 | 1.39 | 1.04 | 0.33 | 0.90 | 0.56 | 1.93 | 0.95 | 0.30 | 0.86 | 0.51 | 1.74 | 0.81 | 0.26 | 0.51 | 0.44 | 1.51 | 1.54 | 0.57 | 0.24 | 0.75 | 3.18 |
| North | 0.88 | 0.27 | 0.69 | 0.49 | 1.61 | 1.06 | 0.32 | 0.84 | 0.59 | 1.93 | 0.92 | 0.28 | 0.78 | 0.51 | 1.66 | 0.65 | 0.20 | 0.16 | 0.36 | 1.19 | 1.65 | 0.59 | 0.16 | 0.82 | 3.33 |
| South | 0.87 | 0.28 | 0.66 | 0.46 | 1.62 | 1.05 | 0.33 | 0.89 | 0.56 | 1.94 | 0.95 | 0.30 | 0.88 | 0.52 | 1.77 | 0.62 | 0.20 | 0.14 | 0.33 | 1.17 | 1.64 | 0.61 | 0.18 | 0.80 | 3.38 |
| **BMI category (base underweight/normal weight)** |  |  |  |  |  |  |  |  |  |  |  |  |  |  |  |  |  |  |  |  |  |  |  |  |  |
| overweight | 0.85 | 0.15 | 0.34 | 0.60 | 1.19 | 0.77 | 0.14 | 0.14 | 0.55 | 1.09 | 1.24 | 0.22 | 0.22 | 0.88 | 1.75 | 1.10 | 0.20 | 0.62 | 0.76 | 1.58 | 1.17 | 0.21 | 0.40 | 0.81 | 1.67 |
| obese | 0.77 | 0.14 | 0.14 | 0.54 | 1.09 | 0.75 | 0.14 | 0.12 | 0.52 | 1.07 | 0.95 | 0.17 | 0.78 | 0.67 | 1.36 | 1.35 | 0.26 | 0.12 | 0.92 | 1.97 | 0.72 | 0.15 | 0.10 | 0.48 | 1.07 |
| **Constant** | 1.27 | 0.49 | 0.53 | 0.60 | 2.70 | 1.33 | 0.51 | 0.46 | 0.63 | 2.80 | 0.65 | 0.25 | 0.26 | 0.31 | 1.37 | 0.86 | 0.34 | 0.69 | 0.39 | 1.86 | 0.20 | 0.09 | <0.01 | 0.08 | 0.47 |
| Obs | 851 |  |  |  |  | 851 |  |  |  |  | 851 |  |  |  |  | 851 |  |  |  |  | 851 |  |  |  |  |
| pseudo R2 | 0.02 |  |  |  |  | 0.01 |  |  |  |  | 0.02 |  |  |  |  | 0.02 |  |  |  |  | 0.04 |  |  |  |  |
| PROB>CHI2 | 0.22 |  |  |  |  | 0.56 |  |  |  |  | 0.19 |  |  |  |  | 0.28 |  |  |  |  | <0.01 |  |  |  |  |

Notes: Multivariate logistic regression. Excludes n=189 respondents for whom weight or height data were missing and BMI could not be calculated.* BMI was not missing at random (those who scored 4-5 were less likely to have BMI missing). Authors' own analysis of Kantar’s Worldpanel Panel Voice survey of 1,040 respondents, November 2022

**Table S4 cont.**

|  | **Support for policies: Information on exercise needed to spend kcal***  **(1 - score 4-5, 0 - score 1-3)** | | | | | **Support for policies:**  **traffic light labels***  **(1 - score 4-5, 0 - score 1-3)** | | | | | **Support for policies:**  **more healthier alternatives on menus***  **(1 - score 4-5, 0 - score 1-3)** | | | | |
| --- | --- | --- | --- | --- | --- | --- | --- | --- | --- | --- | --- | --- | --- | --- | --- |
|  | OR | SE | P>z | 95% CI | | OR | SE | P>z | 95% CI | | OR | SE | P>z | 95% CI | |
| **SES group (base AB)** |  |  |  |  |  |  |  |  |  |  |  |  |  |  |  |
| C1 | 1.01 | 0.2 | 0.94 | 0.69 | 1.49 | 0.95 | 0.17 | 0.77 | 0.67 | 1.35 | 0.96 | 0.17 | 0.83 | 0.67 | 1.37 |
| C2 | 1.37 | 0.32 | 0.18 | 0.87 | 2.16 | 0.74 | 0.16 | 0.17 | 0.48 | 1.14 | 0.84 | 0.18 | 0.43 | 0.55 | 1.29 |
| D | 1.49 | 0.42 | 0.16 | 0.86 | 2.58 | 0.78 | 0.21 | 0.35 | 0.47 | 1.31 | 0.75 | 0.2 | 0.28 | 0.44 | 1.26 |
| E | 0.53 | 0.26 | 0.19 | 0.21 | 1.37 | 0.93 | 0.34 | 0.84 | 0.45 | 1.91 | **0.47** | **0.18** | **0.04** | **0.23** | **0.98** |
| **Sex (base male)** |  |  |  |  |  |  |  |  |  |  |  |  |  |  |  |
| Female | 0.85 | 0.14 | 0.33 | 0.62 | 1.18 | 0.94 | 0.14 | 0.66 | 0.69 | 1.26 | 1.18 | 0.18 | 0.3 | 0.87 | 1.59 |
| **Age group (base under 35 years)** |  |  |  |  |  |  |  |  |  |  |  |  |  |  |  |
| 35-44 | 1.58 | 0.46 | 0.12 | 0.89 | 2.8 | 1.46 | 0.4 | 0.16 | 0.86 | 2.48 | 1.5 | 0.41 | 0.13 | 0.89 | 2.55 |
| 45-54 | 1.53 | 0.45 | 0.15 | 0.86 | 2.71 | 1.53 | 0.41 | 0.12 | 0.9 | 2.59 | 1.44 | 0.39 | 0.17 | 0.85 | 2.45 |
| 55-64 | 1.01 | 0.32 | 0.97 | 0.55 | 1.87 | 1.64 | 0.47 | 0.08 | 0.94 | 2.87 | 1.48 | 0.42 | 0.17 | 0.85 | 2.59 |
| 65+ | 0.56 | 0.2 | 0.1 | 0.28 | 1.12 | 1.07 | 0.32 | 0.83 | 0.59 | 1.93 | 1.49 | 0.45 | 0.19 | 0.83 | 2.69 |
| **Region (base London)** |  |  |  |  |  |  |  |  |  |  |  |  |  |  |  |
| East | 0.69 | 0.23 | 0.27 | 0.36 | 1.34 | 1.39 | 0.45 | 0.31 | 0.74 | 2.61 | 1.5 | 0.48 | 0.21 | 0.8 | 2.83 |
| Midlands | 0.77 | 0.24 | 0.41 | 0.42 | 1.43 | 1.39 | 0.42 | 0.28 | 0.76 | 2.52 | 1.64 | 0.5 | 0.11 | 0.9 | 2.98 |
| North | 0.65 | 0.2 | 0.16 | 0.36 | 1.19 | 0.99 | 0.29 | 0.97 | 0.56 | 1.75 | 1.28 | 0.38 | 0.4 | 0.72 | 2.28 |
| South | 0.58 | 0.19 | 0.09 | 0.31 | 1.09 | 0.99 | 0.3 | 0.97 | 0.54 | 1.8 | 0.94 | 0.29 | 0.84 | 0.52 | 1.71 |
| **BMI category (base underweight/normal weight)** |  |  |  |  |  |  |  |  |  |  |  |  |  |  |  |
| overweight | 1.31 | 0.24 | 0.13 | 0.92 | 1.87 | 1.17 | 0.2 | 0.35 | 0.84 | 1.63 | 1.31 | 0.22 | 0.12 | 0.93 | 1.83 |
| obese | 0.84 | 0.17 | 0.39 | 0.57 | 1.24 | 1.27 | 0.23 | 0.18 | 0.89 | 1.8 | 0.94 | 0.17 | 0.73 | 0.66 | 1.33 |
| **Constant** | 0.55 | 0.22 | 0.13 | 0.25 | 1.19 | 0.71 | 0.27 | 0.36 | 0.34 | 1.48 | 0.63 | 0.24 | 0.22 | 0.3 | 1.31 |
| Obs | 851 |  |  |  |  | 851 |  |  |  |  | 851 |  |  |  |  |
| pseudo R2 | 0.03 |  |  |  |  | 0.01 |  |  |  |  | 0.02 |  |  |  |  |
| PROB>CHI2 | <0.01 |  |  |  |  | 0.30 |  |  |  |  | 0.12 |  |  |  |  |

Notes: Multivariate logistic regression. Excludes n=189 respondents for whom weight or height data were missing and BMI could not be calculated.* BMI was not missing at random (those who scored 4-5 were less likely to have BMI missing). Authors' own analysis of Kantar’s Worldpanel Panel Voice survey of 1,040 respondents, November 2022.

**S5. Participant quotes (as per thematic analysis section)**

**Takeaways as a treat**

*“Most people who order takeaway are not bothered about calories so doubt anything would make a difference”*

*“..by my definition if I elect for a take away it’s not going to be a healthy choice …. I try to minimise take aways and only have one as a special treat”*

*“takeaway is a treat that shouldn't been eaten all the time”*

**Policies targeting the broader food environment**

*“Just make healthy food cheaper so we can all live a healthy life”*

*“Different portion sizes so you can choose the one that fits you”*

*“A wider range of lower calories options rather than just one or two choices”*

*“Having more imaginative healthy options -not just salad! Possibly even with better healthier drink options rather than simply diet versions of fizzy drinks “*

*“it’s the choice of the individual so as long as healthier options are available that is all it should be”*

*“Price is my guide for take-aways, I know they are not healthy. If you make the prices higher, I just won't order in future”*

*I think if people want a takeaway it will almost always be an unhealthy choice and they will just buy less as it gets too expensive. It will really be a choice of yes or no it's too expensive rather than picking something healthier”*

**Policies targeting health education and improving information for making choices**

*“… I would like to see sugar content and fat content in dishes”*

*“The traffic light system is the best. Simple to understand”*

*“Health Warnings - Informing people, they are harming their health and maybe mental wellbeing”*

*“Adverts showing downsides of products that contain a lot of sugar”*

*“I believe more calorie information should be good, I also want more awareness on how much exercise it’ll take to work the food off”*

Source: Authors' own analysis of Kantar’s Worldpanel Panel Voice survey of 1,040 respondents, November 2022
